# Outcomes following penetrating neck injury during the Iraq and Afghanistan conflicts: A comparison of treatment at US and United Kingdom medical treatment facilities

**DOI:** 10.1097/TA.0000000000002625

**Published:** 2020-04-22

**Authors:** John Breeze, Douglas M. Bowley, James G. Combes, James Baden, Linda Orr, Andrew Beggs, Joseph DuBose, David B. Powers

**Affiliations:** From the Royal Centre for Defence Medicine (J. Breeze); University Hospital Birmingham (J. Breeze, D.M.B., J. Baden, L.O.), Birmingham; Royal Surrey County Hospital (J.G.C.), Guildford; Surgical Research Laboratory (A.B.), Institute of Cancer and Genomic Science, University of Birmingham, Birmingham, United Kingdom; Center for the Sustainment of Trauma and Readiness Skills (J.D.), R Adams Cowley Shock Trauma Center, Baltimore, Maryland; and Duke University Medical Center (D.B.P.), Durham, North Carolina.

**Keywords:** Neck, trauma, survival, treatment, military

## Abstract

The United States and United Kingdom (UK) had differing approaches to the surgical skill mix within deployed medical treatment facilities (MTFs) in support of the military campaigns in Iraq and Afghanistan.

**METHODS:**

The US and UK combat trauma registries were scrutinized for patients with penetrating neck injury (PNI) at deployed coalition MTF between March 2003 and October 2011. A multivariate mixed effects logistic regression model (threshold, *p* < 0.05) was used stratified by MTF location and year of injury. The dependent variable was fatality on leaving Role 3, and the independent variables were ISS on arrival, nationality, MTF nationality, and presence of head and neck surgeon.

**RESULTS:**

A total of 3,357 (4.9%) of 67,586 patients who arrived alive at deployed military MTF were recorded to have sustained neck injuries; of which 2,186 (83%) were PNIs and the remainder were blunt injuries. When service members killed in action were included, the incidence of neck injury rose from 4.9% to 10%. Seven hundred nine (32%) of 2,186 patients with PNI underwent neck exploration; 555 patients were recorded to have sustained cervical vascular injury, 230 (41%) of 555 underwent vascular ligation or repair. Where it was recorded, PNI directly contributed to death in 64 (28%) of 228 of patients. Fatality status was positively associated with ISS on arrival (odds ratio, 1.05; 95% confidence interval, 1.04–1.06; *p* < 0.001) and the casualty being a local national (odds ratio, 1.74; 95% confidence interval, 1.28–2.38; *p* < 0.001).

**CONCLUSION:**

Significant differences in the treatment and survival of casualties with PNI were identified between nations in this study; this may reflect differing cervical protection, management protocols, and surgical capability and is worthy of further study. In an era of increasing specialization within surgery, neck exploration remains a skill that must be retained by military surgeons deploying to Role 2 and Role 3 MTF.

**LEVEL OF EVIDENCE:**

Retrospective cohort study, level III.

## Abstract 2

Supplemental digital content is available in the text.

Despite personal protection, combat-associated penetrating neck injury (PNI) remains common and can cause significant long-term morbidity and mortality.^1,2^ Death from PNI is primarily due to airway compromise, exsanguination, or spinal cord trauma,^2,3^ and some deaths after combat PNI are potentially preventable.^4^

Contemporary coalition deployed health care is organized within a coherent network with care provided within echelons or “roles” of care. Role 1 providers deliver specialized first aid, triage, and resuscitation but without surgical capability^5^; topical hemostatic agents can be used in extremity injuries, but their use was not licensed for PNI over this period. An *initial surgical response capability* is provided at Role 2 medical treatment facilities (MTFs); specialized surgery and computed tomography scanning are available at Role 3 MTFs. Role 4 MTFs are in the home nation for United Kingdom (UK) personnel^2,6–8^ and in Germany for US personnel,^9^ with subsequent onward evacuation to Role 5 MTFs in the United States if required.^1,9–11^

In the Role 2 setting in Iraq and Afghanistan for both US and UK MTFs, damage control surgery for PNI was undertaken by general and trauma surgeons; this primarily comprised vascular ligation or repair and surgical airways.^12^ In general, those US and UK surgeons formally trained in the definitive management of PNI were deployed in either Role 2 enhanced or Role 3 facilities, termed in the United States as combat support hospitals or air force theater hospitals.^13^ These included ear, nose, and throat surgeons, trauma surgeons, vascular surgeons, plastic surgeons, and oral and maxillofacial surgeons.

Some casualties underwent tactical aeromedical evacuation directly to a Role 3 MTF, depending on the proximity of the MTF to the point of wounding. For example, in Iraq, the Role 3 MTF in Balad was located in the middle of the so-called *Sunni Triangle*, where approximately 90% of the combat trauma occurred. Patients typically arrived at Role 3 MTF by helicopter within 40-minutes of injury and bypassed Role 2 MTF.^13^ In Afghanistan, more than 90% of casualties were stabilized by surgeons at Roles 2 and 3 MTF by general surgeons alone before tactical aeromedical evacuation transfer to the Role 3 MTF in Bagram.^13^ The main US-led Role 3 hospitals were located at Balad (Iraq), Baghdad (Iraq) and Bagram (Afghanistan).^1,4,9–12,14–19^ The main UK-led Role 3 facilities were Basra (Iraq) and Camp Bastion (Afghanistan).^6–8^ Until 2011, the Canadian-led multi-national Role 3 MTF in Kandahar (Afghanistan) was augmented by clinicians from the US, UK and other nations, including Denmark and Holland,^6–8^ after 2011, it was staffed by US providers.

Previous analyses of PNI from Iraq and Afghanistan have been published, but they enable only limited comparison between US and UK models of care. Some publications are specialty specific and not anatomical region specific,^20^ use surgical logbooks that are subject to epidemiological and reporting bias,^13,15^ and do not describe treatment performed at different deployed facilities.^9^ Finally, the terminology used to describe the *neck* is not always consistent between articles and can include both soft tissue and bone injuries, as well as injuries to the *head and neck*.^9,13,20^ The aim of this analysis was to compare incidence, injury types and treatment performed on US military, UK military, and local civilians during the Iraq and Afghanistan conflicts in order to clarify future surgical training requirements.

## PATIENTS AND METHODS

The US Department of Defense Trauma Registry (US DoDTR) and the UK Joint Theater Trauma Registry (JTTR) were used to identify all patients with PNI sustained between March 1, 2003, and October 31, 2011. The Iraq conflict was described in the databases as Operation Iraqi Freedom (US 2003–2010), Operation New Dawn (US 2010–2011), or Operation TELIC (UK).^7,20^ The Afghanistan conflict was described as Operation Enduring Freedom (US) or Operation HERRICK (UK). The sample size for the US and UK was different, reflecting that troop numbers in Afghanistan in 2011 at the end of this analysis were approximately 90,000 and 9,500 for each country, respectively.^21^ Injuries and treatment were only recorded in those who survived to be admitted to a Role 2 or 3 MTF. Those individuals killed in action (KIA) with PNI were recorded within the UK JTTR alone, as patients KIA were not included in the US DoDTR during this period.

Penetrating neck injury in both registries was matched using AIS codes.^2,17^ The codes used were 300099 and 300999 to 350200. Within the Abbreviated Injury Scale (AIS) system, the neck is body region 3. Treatment performed in deployed US MTF was coded using the *International Classification of Disease, Ninth Revision* (*ICD-9*), codes. Only treatment performed in deployed MTF (Roles 1–3) were included. Treatment performed in deployed UK MTF was coded using the Classification of Interventions and Procedures (OPCS) version 4. Procedures performed were matched to injuries sustained if possible, but not all codes enabled this. No *International Statistical Classification of Disease, 10th Revision*, codes for neck exploration existed, so it was not recorded for US MTF, but OPCS codes include this, and therefore, it was included in the results from UK MTF. For example, in *ICD-9*, there is no procedure code for cervical soft tissue debridement and repair. Codes 86.8 and 86.89 are not specific to the neck and were excluded.

Injury causes were divided into battle, and disease and nonbattle injury. Disease and nonbattle injury comprises accidents, nonhostile incidents, disease, and self-harm. Data were available for US and UK military treated in Kandahar but not for other casualty cohorts. Injury Severity Scores (ISSs) were calculated to demonstrate overall severity.^22^

A multivariate mixed effects logistic regression model (threshold, *p* < 0.05) was used stratified by MTF location and year of injury. The dependent variable was fatality on leaving Role 3, and the independent variables were ISS on arrival (as a continuous variable), nationality, MTF nationality, treatment at Role 2 or 3 without head and neck surgeon, transfer for treatment, and treatment at Role 3 with head and neck surgeon. Odds ratios (ORs) were determined using a χ^2^ test with Yates continuity correction and reported with *p* values and confidence interval. Data analysis was performed using Stata for Mac version 15.1 (StataCorp).

## RESULTS

### Patient Demographics and Injury Mechanisms

**Figure 1:**
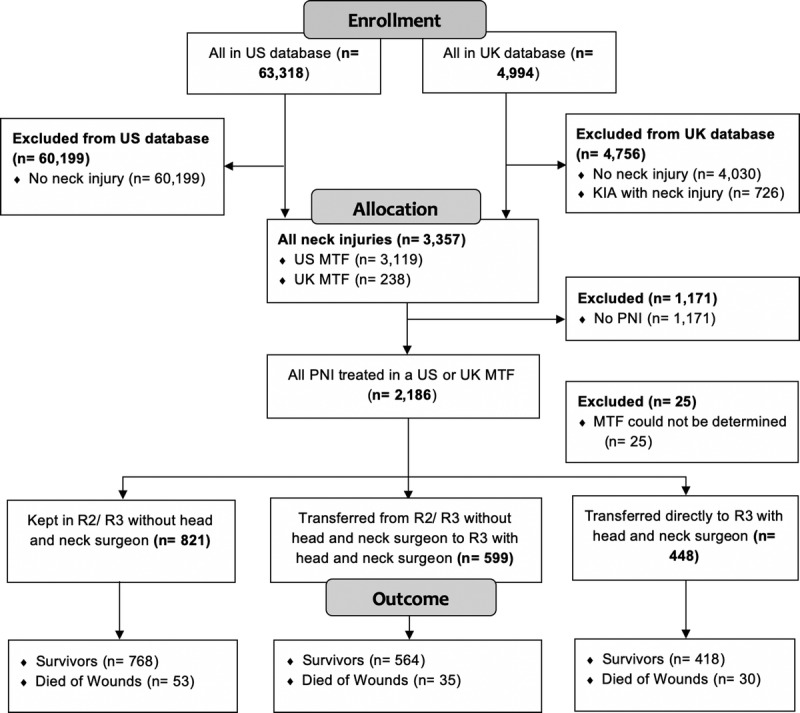
Consolidated Standards of Reporting Trials (CONSORT) flow diagram demonstrating inclusion and exclusion criteria for those who survived to treatment at an MTF.

Neck injuries were found in 3,357 (4.9%) of 67,586 patients who arrived alive at military Role 2 or 3 MTF. Survival outcome was recorded as survivor or died of wounds (DoWs) across both databases (Table 1). Information on those killed in action (KIA) and not surviving to an MTF could not be accurately ascertained from the US data and therefore were not included (Fig. 1). Neck injuries most commonly occurred in battle (3,032 of 3,357 patients, 90%). The most frequent mechanism of injury was explosive devices (2,161 of 3,357 patients, 64%; Supplemental Digital Content 1, Supplementary Table 1, http://links.lww.com/TA/B575). A total of 2,186 (65%) of 3,357 patients were recorded to have sustained PNI (Table 2). The most common cause of PNI was energized fragments from explosive devices (1,260 of 2,186 patients, 58%), followed by gunshot wounds (773 of 2,186 patients, 36%). Of the 2,161 casualties with PNI, 81 (77 US military and 4 UK military) were seen in Kandahar.

**TABLE 1:**
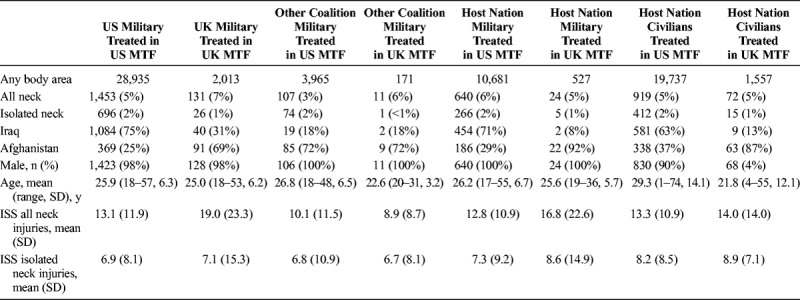
Demographics of Those Casualties With Neck Injuries Who Survived to Treatment at an MTF (SD)

**TABLE 2:**
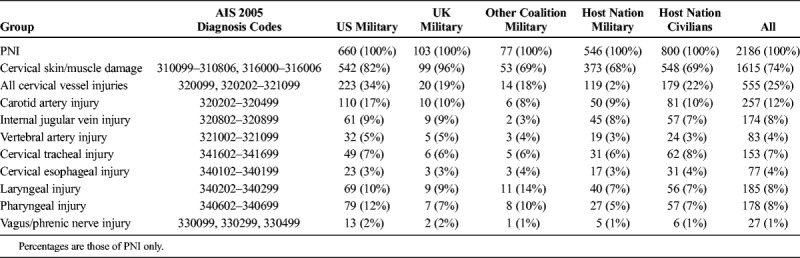
Types of Neck Injuries Found in Those Who Survived to Treatment at a MTF

### Anatomical Causes of Injury

The most common PNIs recorded in casualties who arrived alive at deployed MTF were to skin and/or muscle (1,615 of 2,186 patients, 74%) and major vessels (555 of 2,186 patients, 25%). Of the vascular injuries, the most common recorded vessel injured was the carotid artery (257 of 555 patients, 46%). Airway injuries (laryngeal or tracheal) were found in 307 (14%) of 2,186 PNIs (Table 3). For UK military, when KIA was included as well as DoWs, the incidence of vascular injuries in all neck injuries increased from 15% (20 of 131 cases) to 72% (185 of 258 cases).

**TABLE 3:**
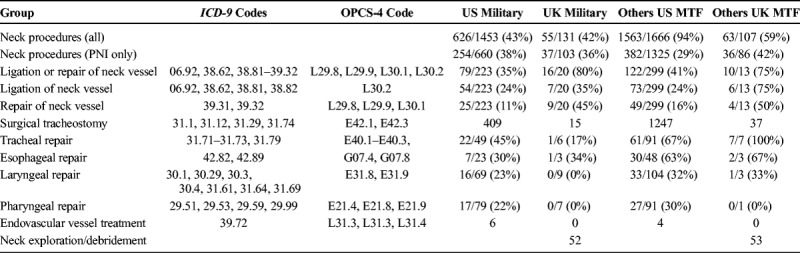
Treatment Performed for PNI in Those Who Survived to Treatment at a MTF

### Treatment Performed as Per Casualty Cohort

Surgery was performed on PNI in 709 (32%) of 2,186 casualties across both databases. Ligation or repair was performed in 230 (41%) of 555 casualties with cervical vessel hemorrhage (Table 3). Endovascular procedures were performed for 10 carotid artery injuries, all of which were performed in US MTF. Cervical airway injuries (laryngeal or tracheal) were treated in 130 (42%) of 307 cases, and pharyngoesophageal injuries were treated in 83 (33%) of 249 cases. No pharyngoesophageal injuries were treated in UK MTF during this period. Surgical tracheostomy was performed in 1,708 (2.5%) of 67,586 patients who arrived alive at deployed MTF having sustained a wound to any body area.

There was no significant difference in the proportion of US military personnel (χ^2^ = 0.151, 1; n = 763; *p* = 0.697; OR, 1.12; confidence interval [CI], 0.73–1.730) having surgery for PNI in deployed MTF compared with UK military personnel. When looking at the US DoDTR alone, US military personnel were significantly more likely (χ^2^ = 18.42, 1; n = 1,985; *p* < 0.0001; OR, 1.54; CI: 1.27–1.88) to have surgery for PNI than other casualty groups. Within the UK JTTR alone, there was no significant difference in the percentage of UK military personnel (χ^2^ = 0.0007, 1; n = 201; *p* = 0.9785; OR, 1.04; CI, 0.59–1.82) having surgery for PNI compared with other casualty groups.

### Treatment Performed by MTF

**Figure 2:**
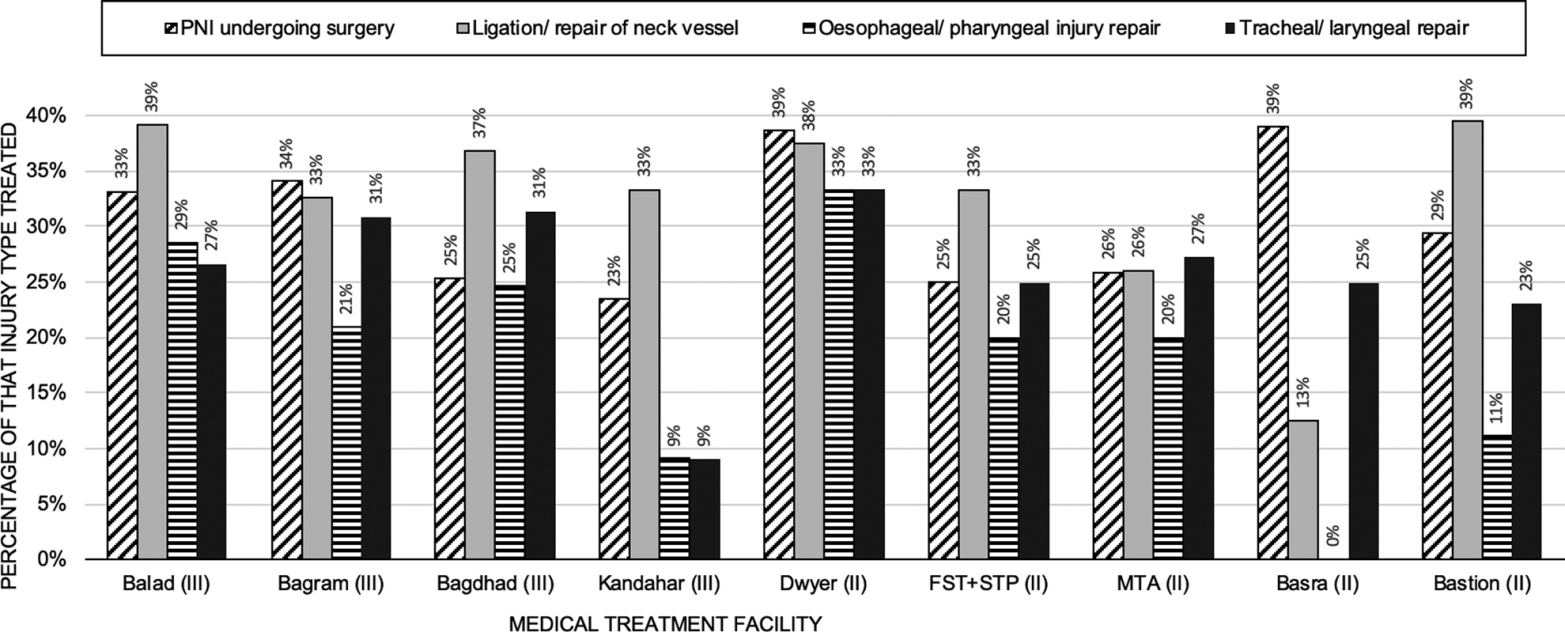
Comparison of treatment performed at different MTFs for casualties with neck injuries that survived to treatment at an MTF.

The highest proportion of PNI treated in casualties who arrived alive at deployed MTF were at Dwyer (12 of 31 patients, 39%) and Basra (16 of 41 patients, 39%; Fig. 2). The MTF least likely to treat US and UK military personnel with PNI was Kandahar (19 of 81 patients, 23%; although this did not reach statistical significance, *p* = 0.2187). When both databases were analyzed together, there was no significant difference in the percentage of casualties with PNI treated surgically at Role 3 MTF (χ^2^ = 0.0302, 1; n = 2065; *p* = 0.697; OR, 1.03; CI, 0.8191–1.292) as compared with Role 2 MTF.

Ligation was more likely to be performed than repair for cervical vascular injuries in US MTF (χ^2^ = 16.6, 1; *p* < 0.001; OR, 1.946; CI, 1.401–2.707). In UK MTF, there was no difference in the proportion of casualties undergoing ligation compared with those undergoing repair of cervical vascular injury (χ^2^ = 0.062, 1; *p* = 0.981; OR, 1.131; CI, 0.382–3.355).

### Association Between PNI and Mortality

Overall, 218 (13%) of 3,357 patients with neck injury died of wounds (Table 1). The UK military personnel with PNI were significantly more likely to have DoWs than US military personnel (χ^2^ = 10.42, 1; n = 763; *p* = 0.0012; OR, 2.77; CI, 1.54–5.09). If patients KIA from the UK JTTR are included, then neck wounds were found in 361 (13%) of 3,502 patients who died (Table 4). The relationship between neck wound and death could be ascertained in 57 (30%) of 193 fatalities in the US DoDTR (DoWs) and 162 (100%) of 162 fatalities (KIA + DoWs) in the UK JTTR (Table 4). In the UK JTTR, 35 (9%) of 383 neck wounds were believed to be directly contributory to death (KIA or DoWs). Of the 65 casualties who DoWs with neck injuries but the neck itself was not the cause of death, 42 (65%) of 65 deaths were due to head injury.

**TABLE 4:**
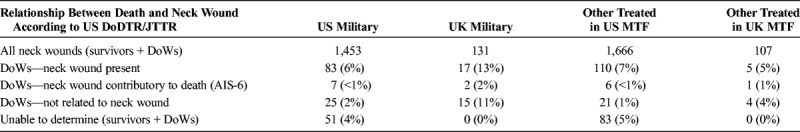
Association Between Neck Wound and Fatalities Based Upon Casualty Cohort

Across both databases, 66 (9%) of 773 casualties with PNI from Gunshot Wound (GSW) and 74 (6%) of 1,260 casualties with PNI from an explosive event died of wounds. Considering all casualties with neck wounds, death was statistically more likely (χ^2^ = 10.42, 1; n = 2,033; *p* = 0.0269; OR, 1.49; CI, 1.06–2.10) after GSW than energized fragments from explosive devices (Supplemental Digital Content 1, Supplementary Table 1, http://links.lww.com/TA/B575). In US and UK military personnel who DoWs after PNI, the dominant cervical injury was vascular (62 of 589 patients, 11%) followed by airway (trachea/larynx) injury (14 of 284 patients, 5%).

### Association Between Mortality of Those With PNI and MTF

Across both databases, PNI treated at Role 2 MTF were more likely to die (χ^2^ = 41.19, 1; n = 2,065; *p* = <0.0001; OR, 3.08; CI, 2.17–4.37) than casualties with PNI treated at Role 3 MTF. Casualties with PNI were statistically more likely (χ^2^ = 11.89, 1; n = 1,967; *p* = 0.0006; OR, 2.18; CI, 1.41–3.34) to have died in UK MTF than US MTF.

### Results of Regression Analysis

When analyzing results from both the US and UK databases combined, fatality status was positively associated with ISS on arrival (OR, 1.05; 95% CI, 1.04–1.06; *p* < 0.001) and the casualty being a local national (OR, 1.74; 95% CI, 1.28–2.38; *p* < 0.001). There was no significant effect of a head and neck surgeon being present or whether the MTF was US or UK led (Supplemental Digital Content 2, Supplementary Table 2, http://links.lww.com/TA/B576). As measured by area under the curve, the model had a reasonably good fit for the observed data (area under the curve, 0.73; 95% CI, 0.70–0.76).

## DISCUSSION

Combat-associated neck injury remains relatively common and associated with significant morbidity and mortality; in this series, overall, PNIs were recorded in 5% of casualties who arrived alive at deployed MTF. Combat-associated PNI is a highly morbid injury; when service members KIA were included, the incidence of neck injury rose from 5% to 10%. This project is, to our knowledge, the first to directly compare patterns of neck injury and treatments undertaken in deployed US and the UK MTF.

The most common causes of PNI were energized fragments from explosive devices (64%) and GSW (23%). Improvised explosive devices produce multiple fragments that pepper the exposed neck^23^ and, despite the relatively innocuous entry wounds, can produce extensive underlying injury. This pattern of wounding has led some US authors to recommend mandatory surgical exploration of such wounds.^11^ In our article, 9% of casualties with cervical GSW and 6% of those injured by energized fragments were recorded within the trauma registries as having DoW, a higher observed mortality rate than the single surgeon series by Brennan et al.^13^ that reported mortality rates of 1.3% to 5.3% from PNIs in Iraq and Afghanistan. Previous UK JTTR and postmortem studies that include KIA^2,24,25^ have also reported high mortality rates; for example, Stannard et al.^25^ determined that 13 (76%) of 17 UK military personnel sustaining cervical vascular trauma and treated in a UK MTF died and the postmortem analysis of combat neck injury by Breeze et al.^2^ found that the mortality of patients injured by energized fragments to the neck was 41% and the mortality after GSW was 78% if the entry wound was the neck. When analyzing KIA and DoW data from the UK JTTR in our study, patients injured by GSW had a mortality of 47%, and patients with PNI from energized fragments had a mortality of 37%.

With the exception of superficial injuries, the most common neck injuries were to major vessels (17%). A similar rate of vascular injuries has been identified in previous analyses^26,27^ and likely reflects the vulnerable position of the carotid arteries and jugular veins in the neck.^2^ More carotid than Internal Jugular Vein (IJV) vascular injuries were recorded, despite the latter having a more superficial location in the neck; this may reflect underrecording of IJV injuries, particularly on computed tomography scans. Otherwise, no other predominant anatomical injury patterns were obvious from the data. Surgical ligation or repair was performed in 41% of casualties with cervical vessel hemorrhage; ligation was more commonly undertaken than repair in US MTF, but repair was equally likely to be performed compared with ligation in UK MTF.^15,18^ The relatively low number of recorded vertebral artery injuries treated in an MTF (15% of cervical vessel injuries) most likely reflects the greater protection that the vertebral arteries have to penetrative injury because of their anatomical position within the foramen transversarium of the cervical vertebrae for the majority of their course.^2,28^ It may also reflect that these injuries may be more difficult to identify clinically and certainly to treat operatively, and low numbers may reflect underreporting.

Cervical airway injuries (laryngeal or tracheal) were found in 14% of PNIs. Such injuries have high mortality, with an analysis of postmortem records from the UK military ascribing them as the cause of death in 79% of cases of PNIs.^24^ Forty-two percent of laryngotracheal injuries were treated surgically. The indications for acute interventions in laryngotracheal injury are controversial, with an experienced anesthetist often capable of intubating complex penetrating trauma. Brennan et al.^1^ described 25 surgical interventions for acute interventions in laryngotracheal injury for 6 years in Balad, basing indications for surgery on those described in the civilian literature by Schaefer et al.^14^ Pharygoesophageal injuries were found in only 11% of PNIs in this study, similar to comparable analyses.^24^ Again, this relatively low incidence may represent inherent anatomical shielding by superficial structures or underrecording of injuries potentially due to difficulty in diagnosis. An analysis of clinical outcomes from 17 UK soldiers who sustained pharyngoesophageal injuries demonstrated that injuries were almost always associated with other devastating injuries with either immediate or very early fatal outcome.^24^ The treatment of these injuries can be more challenging than those of the larynx and trachea, yet only one third was surgically treated in the Roles 2 and 3 setting. However, injuries to the pharynx and cervical esophagus that are missed can result in significant morbidity.^24^ Currently, there is little evidence to support definitive treatment of such wounds before evacuation to Role 4 either in Germany or the United Kingdom, but recognition and control of soiling from the injured organs remain paramount.

Significant differences were found in the likelihood of certain injury types being treated between casualty cohorts. The groups most likely to have surgical intervention of PNI were host nation civilians or military in UK MTF (42%) and those least likely were host nation civilians or military in US MTF (29%). The high incidence in noncoalition military casualties reflected that for many this was definitive surgery and close monitoring required for conservative management was unlikely to be available in the local health care system. Although, overall, US military personnel with PNI were significantly more likely to undergo neck exploration (OR, 3.0) than UK military personnel, this was highly variable between individual surgeons. The proportions of PNI treated varied over time at all locations. Casualties with PNI treated at Role 3 MTF were 3 times more likely to survive than those in a Role 2 MTF, despite being equally likely to receive surgical intervention. This finding was the same when the US DoDTR was analyzed alone. Multiple reasons for this may exist; Role 3 MTFs were perhaps more likely to have surgeons formally trained in the treatment of PNIs. However, it may also reflect that the most severely wounded were treated at Role 2.

The US and UK military body armor provided generally similar ballistic protection; however, US armor differed because the collars were integral to their body armor, while the UK's was removable. In a review of UK military neck injuries from 2011, ballistic cervical protection was found to have prevented penetration of energized fragments from explosive devices on numerous occasions.^2^ However, a lower rate of wearing of neck protection by UK military personnel in Afghanistan was identified compared with their US counterparts.^2^ This was reported as being due to troop discomfort and perceived difficulty with equipment integration reported as the main constrain to routine use.^29^ Postmortem analysis identified that the use of neck collars could have potentially prevented 16 (10.5%) of 152 deaths of UK service personnel sustaining neck wounds had they been worn.^2^ The UK military neck collars were redesigned, and the new type issued from 2013 onwards and the wearing of collars by troops on foot patrol and static sentry positions were mandated.^28^ However, since the UK withdrew from Afghanistan shortly after 2013, no change in incidence or severity of UK neck injury post 2013 can be estimated to ascertain the effectiveness of the new equipment and policy.

The United States and United Kingdom have previously collaborated in terms of surgical deployments, and this is likely to continue in the future. However, optimal functioning of the surgical teams relies on each country understanding the skill sets of the surgeons that they are deploying to optimize interoperability. This is made more difficult by the evolution of surgical training, with ever greater subspecialization and differences in the terminology and training of specialties between countries. The largest proportion of potentially survivable neck wounds is vascular in origin.^2,18,19^ In the United Kingdom, general and vascular surgery are now distinct surgical specialties with separate training programs. The deployed general surgeon may or may not have a vascular background, depending on training and the requirements of their substantive consultant appointment within the UK National Health Service.^25^ A study from 2010 demonstrated that, by the end of their training, US general surgical residents had, on average, performed only 3 neck explorations.^30^ The average US general surgery chief resident graduating in 2016 had only performed 2.5 surgical procedures for vascular trauma.^27^ As a result of these shifts in training, the military may have fewer surgeons who can hold *dual roles* (i.e., general surgeons who can perform vascular surgery, vascular surgeons who can perform general surgery, and cardiothoracic surgeons who can perform general surgery).^27^ This will result in the potential need to deploy both vascular and general surgeons. The UK military in particular need to consider deploying head and neck surgeons, similar to that undertaken by the US military.^10–13^

The current study has several limitations, including its retrospective nature and the requirement to exclude US data on KIA because of incomplete capture at Role 2 during this period.^31^ Some casualties who were KIA were never brought to either a Role 2 or Role 3 facility, and their numbers cannot therefore be accurately determined. It is possible that more severely injured casualties in the US medical system were taken to Role 2 first instead of Role 3, thereby skewing the results in particular those who DoWs. The US DoDTR could not determine the location of surgery of those treated at both Role 2 and Role 3, only that surgery occurred. Data capture relies on the accuracy and completeness of medical records, which can be challenging depending on the logistical and clinical situation at deployed MTF. It is possible that the higher incidence of neck injuries seen in US MTF reflected either greater recording of injuries or suboptimal recording at UK MTF. The US DoDTR did not record neck exploration as a separate code, which has been reported as the third most common procedure for PNI.^12,13^ Repeat procedures with the same *ICD* or OPCS codes and performed on the same patient at the same MTF are documented only once. Care must be taken in the interpretation of these findings because the mechanism of injury recorded by the clinicians was sometimes their own personal interpretation of the medical record available to them, which was not always complete. As with other JTTR analyses, some procedures were classified as unspecified because the *International Classification of Diseases, Ninth Revision, Clinical Modification*, code did not fall into a procedure skill set.^27^ For example, “39.98, control of hemorrhage, NOS,” was too nonspecific to categories into any anatomic region or procedure category and may refer to either vascular or nonvascular procedures (e.g., packing) but were included in the analysis for completeness.^27^ Finally, the overall differences in incidence and mortality associated with these neck injuries may reflect that there was greater recording of minor injuries in the US MTF and underrecording of mortality, particularly at Role 2.^31^

In conclusion, in this study, casualties with PNI treated at US MTF were significantly more likely to survive than casualties treated at UK MTF, despite being equally likely to receive treatment. In addition, the odds of survival for casualties with PNI treated at Role 3 MTF were 3 times higher than those in a Role 2 MTF, despite being equally likely to receive treatment. This may reflect that Role 3 MTFs were more likely to have surgeons formally trained in the treatment of PNIs. We believe that the results of this article support previous multidisciplinary military consensus that neck exploration is an essential skill that must be retained by those surgeons deploying to coalition Roles 2 and 3 MTF in future conflicts.^32^

## Supplementary Material

SUPPLEMENTARY MATERIAL
