# Supplementary material for: Outcomes following penetrating neck injury during the Iraq and Afghanistan conflicts: A comparison of treatment at US and United Kingdom medical treatment facilities
Source: J Trauma Acute Care Surg. 2020 Apr 22;88(5):696–703. doi: 10.1097/TA.0000000000002625 (PMC7182242; doi:10.1097/TA.0000000000002625)
Supplement: SUPPLEMENTARY MATERIAL [file ta-88-696-s001.docx]

**Introduction**

Despite personal protection, combat-associated penetrating neck injury (PNI) remains common and can cause significant long-term morbidity and mortality ^1,2^. Death from PNI is primarily due to airway compromise, exsanguination, or spinal cord trauma ^2,3^ and some deaths after combat PNI are potentially preventable ^4^.

Contemporary coalition deployed healthcare is organized within a coherent network with care provided within echelons or “roles” of care. Role 1 providers deliver specialized first aid, triage, and resuscitation, but without surgical capability ^5^; topical hemostatic agents can be utilized in extremity injuries but their use was not licensed for PNI over this time period. An ‘initial surgical response capability’ is provided at Role 2 MTF; specialized surgery and CT scanning is available at R3 MTF. R4 MTF are in the home nation for UK personnel ^2,6–8^, and in Germany for US personnel ^9^, with subsequent onward evacuation to R5 MTF in the US if required ^1,9–11^.

In the Role 2 setting in Iraq and Afghanistan for both US and UK MTF, damage control surgery for PNI was undertaken by general and trauma surgeons; this primarily comprised vascular ligation or repair and surgical airways ^12^. In general, those US and UK surgeons formally trained in the definitive management of PNI were deployed in either Role 2 Enhanced or Role 3 facilities, termed in the US as Combat Support Hospitals, or Air Force Theater Hospitals ^13^. These included Ear Nose and Throat surgeons, trauma surgeons, vascular surgeons, Plastic surgeons, and Oral and Maxillofacial (OMF) surgeons.

Some casualties underwent tactical aeromedical evacuation (TACEVAC) directly to a Role 3 MTF, depending on the proximity of the MTF to the point of wounding. For example, in Iraq, the Role 3 MTF in Balad was located in the middle of the so-called ‘Sunni Triangle’, where approximately 90% of the combat trauma occurred. Patients typically arrived at Role 3 MTF by helicopter within 40-minutes of injury and bypassed Role 2 MTF ^13^. In Afghanistan, >90% of casualties were stabilized by surgeons at Role 2 and 3 MTF by general surgeons alone before TACEVAC transfer to the Role 3 MTF in Bagram ^13^. The main US-led Role 3 hospitals were located at Balad (Iraq), Baghdad (Iraq) and Bagram (Afghanistan) ^1,4,18,19,9–12,14–17^. The main UK-led Role 3 facilities were Basra (Iraq) and Camp Bastion (Afghanistan) ^6–8^. Until 2011, the Canadian-led multi-national Role 3 MTF in Kandahar (Afghanistan) was augmented by clinicians from the US, UK and other nations, including Denmark and Holland ^6–8^, after 2011, it was staffed by US providers.

Previous analyses of PNI from Iraq and Afghanistan have been published, but they enable only limited comparison between US and UK models of care. Some publications are specialty specific and not anatomical region specific ^20^, utilize surgical logbooks that are subject to epidemiological and reporting bias ^13,15^ and do not describe treatment performed at different deployed facilities ^9^. Finally, the terminology used to describe the ‘neck’ is not always consistent between papers, and can include both soft tissue and bone injuries, as well as injuries to the ‘head and neck’ ^9,13,20^. The aim of this analysis was to compare incidence, injury types and treatment performed on US military, UK military and local civilians during the Iraq and Afghanistan conflicts in order to clarify future surgical training requirements.

**Method**

The US Department of Defense Trauma Registry (US DoDTR) and the UK Joint Theatre Trauma Registry (JTTR) were used to identify all patients with PNI sustained between 01 March 2003 and 31 October 2011. The Iraq conflict was described in the databases as Operation Iraqi Freedom (US 2003-2010), Operation New Dawn (US 2010-2011) or Operation TELIC (UK) ^7,20^. The Afghanistan conflict was described as Operation Enduring Freedom (US) or Operation HERRICK (UK). The sample size for the US and UK was different, reflecting that troop numbers in Afghanistan in 2011 at the end of this analysis was approximately 90,000 and 9,500 for each country respectively ^21^. Injuries and treatment were only recorded in those who survived to be admitted to a Role 2 or 3 MTF. Those individuals Killed in Action (KIA) with PNI were recorded within the UK JTTR alone, as patients KIA was not included in the US DoDTR during this period.

PNI in both registries were matched using AIS codes ^2,17^. The codes used were: 300099, 300999- 350200. Within the AIS system, the neck is body region 3. Treatment performed in deployed US MTF was coded using the International Classification of Disease version 9 (ICD-9) codes. Only treatment performed in deployed MTF (Roles 1- 3) were included. Treatment performed in deployed UK MTF was coded using the Classification of Interventions and Procedures (OPCS) version 4. Procedures performed were matched to injuries sustained if possible but not all codes enabled this. No ICD-10 codes for neck exploration existed so it was not recorded for US MTF but OPCS codes include this and therefore it was included in the results from UK MTF. For example, in ICD 9 there is no procedure code for cervical soft tissue debridement and repair. Codes 86.8 and 86.89 are not specific to the neck and were excluded.

Injury causes were divided into battle and disease non-battle (DNBI). DNBI comprises accidents, non-hostile incidents, disease and self-harm. Data were available for US and UK military treated in Kandahar, but not for other casualty cohorts. Injury Severity Scores (ISS) were calculated to demonstrate overall severity ^22^.

A multivariate mixed effects logistic regression model (threshold p< 0.05) was used stratified by MTF location and year of injury. The dependent variable was fatality on leaving Role 3, and independent variables were ISS on arrival (as a continuous variable), nationality, MTF nationality, treatment at Role 2 or 3 without head and neck surgeon, transfer for treatment and treatment at Role 3 with head and neck surgeon. Odds ratios were determined using a Chi-Square test with Yates’ continuity correction, and reported with p-values and confidence interval. Data analysis was performed using Stata for Mac version 15.1 (StataCorp, USA).

**Results**

***Patient demographics and injury mechanisms***

Neck injuries were found in 3357/67586 (4.9%) of patients who arrived alive at military R2/3 MTF. Survival outcome was recorded as survivor or died of wounds (DoW) across both databases (Table 1). Information on those Killed in Action (KIA) and not surviving to an MTF could not be accurately ascertained from the US data and therefore were not included (Figure 1). Neck injuries most commonly occurred in battle (3032/3357, 90%). The most frequent mechanism of injury was explosive devices 2161/3357 (64%, Supplemental Digital Content 1, Table 1, <http://links.lww.com/TA/B575>). 2186/3357 (65%) were recorded to have sustained PNI (Table 2). The most common causes of PNI was energized fragments from explosive devices 1260/2186 (58%), followed by gunshot wounds 773/2186 (36%). Of the 2161 casualties with PNI, 81 (77 US military and 4 UK military) were seen in Kandahar.

***Anatomical causes of injury***

The most common PNI recorded in casualties who arrived alive at deployed MTF were to skin and/or muscle (1615/2186, 74%), and major vessels (555/2186, 25%). Of the vascular injuries, the most common recorded vessel injured was the carotid artery (257/555, 46%). Airway injuries (laryngeal or tracheal) were found in 307/2186 (14%) of PNI (Table 3). For UK military, when KIA was included as well as DoW, the incidence of vascular injuries in all neck injuries increased from 20/131 (15%) to 185/258 (72%).

***Treatment performed as per casualty cohort***

Surgery was performed on PNI in 709/2186 (32%) of casualties across both databases. Ligation or repair was performed in 230/555 (41%) of casualties with cervical vessel hemorrhage (Table 3). Endovascular procedures were performed for 10 carotid artery injuries, all of which were performed in US MTF. Cervical airway injuries (laryngeal or tracheal) were treated in 130/307 (42%) cases and pharyngo-esophageal injuries treated in 83/249 (33%) cases. No pharyngo-esophageal injuries were treated in UK MTF during this period. Surgical tracheostomy was performed in 1708/67586 (2.5%) patients who arrived alive at deployed MTF having sustained a wound to any body area.

There was no significant difference in the proportion of US military personnel (X^2^ 0.151, 1. N=763, p= 0.697, OR: 1.12, CI: 0.73-1.730) having surgery for PNI in deployed MTF compared to UK military personnel. When looking at the US DoDTR alone, US military personnel were significantly more likely (X^2^ 18.42, 1. N=1985, p<0.0001, OR: 1.54, CI: 1.27-1.88) to have surgery for PNI than other casualty groups. Within the UK JTTR alone, there was no significant difference in the percentage of UK military personnel (X^2^ 0.0007, 1. N=201, p=0.9785, OR: 1.04, CI: 0.59 -1.82) having surgery for PNI compared to other casualty groups.

***Treatment performed by Medical Treatment Facility***

The highest proportion of PNI treated in casualties who arrived alive at deployed MTF were at Dwyer (12/31, 39%) and Basra (16/41, 39%, Figure 2). The MTF least likely to treat US and UK military personnel with PNI was Kandahar (19/81, 23%, although this did not reach statistical significance p=0.2187). When both databases were analyzed together, there was no significant difference in the percentage of casualties with PNI treated surgically at Role 3 MTF (X^2^ 0.0302, 1. N=2065, p= 0.697, OR: 1.03, CI: 0.8191-1.292) than Role 2 MTF.

Ligation was more likely to be performed than repair for cervical vascular injuries in US MTF (X^2^ 16.6, 1. P<0.001, OR: 1.946, CI: 1.401-2.707). In UK MTF, there was no difference in the proportion of casualties undergoing ligation compared to those undergoing repair of cervical vascular injury (X^2^ 0.062, 1. P= 0.981, OR: 1.131, CI: 0.382-3.355).

***Association between PNI and mortality***

Overall, 218/3357 (13%) of patients with neck injury DoW (Table 1). UK military personnel with PNI were significantly more likely to have DoW than US military personnel (X^2^ 10.42, 1. N=763, p= 0.0012, OR: 2.77, CI: 1.54-5.09). If KIA from the UK JTTR are included, then neck wounds were found in 361/3502 (13%) of those who died (Table 4). The relationship between neck wound and death could be ascertained in 57/193 (30%) fatalities in the US DoDTR (DoW) and 162/162 (100%) fatalities (KIA + DoW) in the UK JTTR (Table 4). In the UK JTTR, 35/383 (9%) of neck wounds were believed to be directly contributory to death (KIA or DoW). Of the 65 casualties that DoW with neck wounds but the neck itself was not the cause of death, 42/65 (65%) of deaths were due to head injury.

Across both databases, 66/773 (9%) of casualties with PNI from GSW and 74/1260 (6%) with PNI from an explosive event DoW. Considering all casualties with neck wounds, death was statistically more likely (X^2^ 10.42, 1. N=2033, p= 0.0269, OR: 1.49, CI: 1.06-2.10) after GSW than energized fragments from explosive devices (Supplemental Digital Content 1, Table 1, <http://links.lww.com/TA/B575>). In US and UK military personnel who DoW after PNI, the dominant cervical injury was vascular (62/589, 11%) followed by airway (trachea/larynx) injury (14/284, 5%).

***Association between mortality of those with PNI and Medical Treatment Facility***

Across both databases, PNI treated at Role 2 MTF were more likely to die (X^2^ 41.19, 1. N=2065, p= <0.0001, OR: 3.08, CI: 2.17 to 4.37) than casualties with PNI treated at Role 3 MTF. Casualties with PNI were statistically more likely (X^2^ 11.89, 1. N=1967, p= 0.0006, OR: 2.18, CI: 1.41- 3.34) to have died in UK MTF than US MTF.

***Results of regression analysis***

When analyzing results from both the US and UK databases combined, fatality status was positively associated with ISS on arrival (OR 1.05, 95% CI 1.04 -1.06, p<0.001) and the casualty being a local national (OR 1.74, 95% CI: 1.28-2.38, p<0.001). There was no significant effect of a Head and Neck surgeon being present, or whether the MTF was US or UK led (Supplemental Digital Content 2, Table 2, <http://links.lww.com/TA/B576>). As measured by AUC, the model had a reasonably good fit for the observed data (AUC 0.73, 95% CI 0.70-0.76).

**Discussion**

Combat-associated neck injury remains relatively common and associated with significant morbidity and mortality; in this series overall, PNI were recorded in 5% of casualties who arrived alive at deployed MTF. Combat-associated PNI is a highly morbid injury; when service members KIA were included, the incidence of neck injury rose from 5% to 10%. This project is, to our knowledge, the first to directly compare patterns of neck injury and treatments undertaken in deployed US and the UK MTF.

The most common causes of PNI was energized fragments from explosive devices (64%), and GSW (23%). Improvised explosive devices (IED) produce multiple fragments that pepper the exposed neck ^23^, and despite the relatively innocuous entry wounds, can produce extensive underlying injury. This pattern of wounding has led some US authors to recommend mandatory surgical exploration of such wounds ^11^. In our paper, 9% of casualties with cervical GSW and 6% of those injured by energized fragments were recorded within the trauma registries as having DOW, a higher observed mortality rate than the single surgeon series by Brennan *et al.* that reported mortality rates of 1.3%-5.3% from PNI in Iraq and Afghanistan ^13^. Previous UK JTTR and post mortem studies that include KIA ^2,24,25^ have also reported high mortality rates; for example, Stannard *et al.* determined that 13/17 (76%) of UK military personnel sustaining cervical vascular trauma and treated in a UK MTF died ^25^ and the post mortem analysis of combat neck injury by Breeze *et al.* that found the mortality of patients injured by energized fragments to the neck was 41% and the mortality after GSW was 78% if the entry wound was the neck ^2^. When analyzing KIA and DOW data from the UK JTTR in our study, patients injured by GSW had a mortality of 47%, and patients with PNI from energized fragments had a mortality of 37%.

With the exception of superficial injuries, the most common neck injuries were to major vessels (17%). A similar rate of vascular injuries has been identified in previous analyses ^26,27^, and likely reflects the vulnerable position of the carotid arteries and jugular veins in the neck ^2^. More carotid than IJV vascular injuries were recorded, despite the latter having a more superficial location in the neck; this may reflect under-recording of IJV injuries, particularly on CT scans. Otherwise no other predominant anatomical injury patterns were obvious from the data. Surgical ligation or repair was performed in 41% of casualties with cervical vessel hemorrhage; ligation was more commonly undertaken than repair in US MTF but repair was equally likely to be performed compared to ligation in UK MTF^15,18^. The relatively low number of recorded vertebral artery injuries treated in an MTF (15% of cervical vessel injuries) most likely reflects the greater protection that the vertebral arteries have to penetrative injury because of their anatomical position within the foramen transversarium of the cervical vertebrae for the majority of their course ^2,28^. It may also reflect that these injuries may be more difficult to identify clinically and certainly to treat operatively and low numbers may reflect under-reporting.

Cervical airway injuries (laryngeal or tracheal) were found in 14% of PNI. Such injuries have high mortality, with an analysis of post mortem records from the UK military ascribing them as the cause of death in 79% of cases of PNI ^24^. Forty-two percent of laryngotracheal injuries were treated surgically. The indications for acute interventions in laryngotracheal injury are controversial, with an experienced anesthetist often capable of intubating complex penetrating trauma. Brennan *et al.* described 25 surgical interventions for acute interventions in laryngotracheal injury over 6 years in Balad ^1^, basing indications for surgery on those described in the civilian literature by Schaefer *et al.* ^14^. Pharygo-esophageal injuries were found in only 11% of PNI in this study, similar to comparable analyses ^24^. Again, this relatively low incidence may represent inherent anatomical shielding by superficial structures or under recording of injuries potentially due to difficulty in diagnosis. An analysis of clinical outcomes from 17 UK soldiers who sustained pharyngo-esophageal injuries, demonstrated that injuries were almost always associated with other devastating injuries with either immediate or very early fatal outcome ^24^. The treatment of these injuries can be more challenging than those of the larynx and trachea, yet only one third were surgically treated in the Role 2 and 3 setting. However, injuries to the pharynx and cervical esophagus that are missed can result in significant morbidity ^24^. Currently there is little evidence to support definitive treatment of such wounds before evacuation to Role 4 either in Germany or the UK, but recognition and control of soiling from the injured organs remains paramount.

Significant differences were found in the likelihood of certain injury types being treated between casualty cohorts. The groups most likely to have surgical intervention of PNI were host nation civilians or military in UK MTF (42%) and those least likely were host nation civilians or military in US MTF (29%). The high incidence in non-coalition military casualties reflected that for many this was definitive surgery and close monitoring required for conservative management was unlikely to be available in the local healthcare system. Although overall, US military personnel with PNI were significantly more likely to undergo neck exploration (OR 3.0) than UK military personnel, this was highly variable between individual surgeons. The proportions of PNI treated varied over time at all locations. Casualties with PNI treated at Role 3 MTF were three times more likely to survive than those in a Role 2 MTF, despite being equally likely to receive surgical intervention. This finding was the same when the DoDTR was analyzed alone. Multiple reasons for this may exist; Role 3 MTF were perhaps more likely to have surgeons formally trained in the treatment of PNI. However, it may also reflect that the most severely wounded were treated at Role 2.

US and UK military body armor provided generally similar ballistic protection; however, US armor differed as the collars were integral to their body armor, whilst the UK’s was removable. In a review of UK military neck injuries from 2011, ballistic cervical protection was found to have prevented penetration of energized fragments from explosive devices on numerous occasions ^2^. However, a lower rate of wearing of neck protection by UK military personnel in Afghanistan was identified compared to their US counter-parts ^2^. This was reported as being due to troop discomfort and perceived difficulty with equipment integration reported as the main constrain to routine use ^29^. Post mortem analysis identified that the use of neck collars could have potentially prevented 16/152 (10.5%) deaths of UK service personnel sustaining neck wounds had they been worn ^2^. The UK military neck collars were redesigned and the new type issued from 2013 onwards and the wearing of collars by troops on foot patrol and static sentry positions was mandated ^28^. However, since the UK withdrew from Afghanistan shortly after 2013, no change in incidence or severity of UK neck injury post 2013 can be estimated to ascertain the effectiveness of the new equipment and policy.

The US and UK have previously collaborated in terms of surgical deployments and this is likely to continue in the future. However, optimal functioning of the surgical teams relies on each country understanding the skill sets of the surgeons that they are deploying to optimize interoperability. This is made more difficult by the evolution of surgical training, with ever greater sub-specialization and differences in the terminology and training of specialties between countries. The largest proportion of potentially survivable neck wounds are vascular in origin ^2,18,19^. In the UK, general and vascular surgery are now distinct surgical specialties with separate training programs. The deployed general surgeon may or may not have a vascular background, depending on training and the requirements of their substantive consultant appointment within the UK National Health Service ^25^. A study from 2010 demonstrated that, by the end of their training, US general surgical residents had, on average, performed only 3 neck explorations ^30^. The average US general surgery chief resident graduating in 2016 had only performed 2.5 surgical procedures for vascular trauma ^27^. As a result of these shifts in training, the military may have fewer surgeons who can hold “dual roles” (i.e. general surgeons who can perform vascular surgery, vascular surgeons who can perform general surgery, and cardiothoracic surgeons who can perform general surgery) ^27^. This will result in the potential need to deploy both vascular and general surgeons. The UK military in particular need to consider deploying head and neck surgeons, similar to that undertaken by the US military ^10–13^.

The current study has several limitations, including its retrospective nature and the requirement to exclude US data on KIA due to incomplete capture at Role 2 during this period ^31^. Some casualties who were KIA were never brought to either a R2 or R3 facility and their numbers cannot therefore be accurately determined. It is possible that more severely injured casualties in the US medical system were taken to R2 first instead of R3, thereby skewing the results in particular those who DoW. The DoDTR could not determine the location of surgery of those treated at both R2 and R3, only that surgery occurred. Data capture relies on the accuracy and completeness of medical records, which can be challenging depending on the logistical and clinical situation at deployed MTF. It is possible that the higher incidence of neck injuries seen in US MTF reflected either greater recording of injuries or sub-optimal recording at UK MTF. The US DoDTR did not record neck exploration as a separate code, which has been reported as the third most common procedure for PNI ^12,13^. Repeat procedures with the same ICD or OPCS codes and performed on the same patient at the same MTF are documented only once. Care must be taken in the interpretation of these findings as the mechanism of injury recorded by the clinicians was sometimes their own personal interpretation of the medical record available to them, which was not always complete. As with other JTTR analyses, some procedures were classified as unspecified because the ICD-9-CM code did not fall into a procedure skill set ^27^. For example, “39.98, control of hemorrhage, NOS,” was too nonspecific to categories into any anatomic region or procedure category and may refer to either vascular or nonvascular procedures (e.g. packing), but were included in the analysis for completeness ^27^. Finally, the overall differences in incidence and mortality associated with these neck injuries may reflect that there was greater recording of minor injuries in the US MTF, and under recording of mortality, particularly at Role 2 ^31^.

In conclusion, in this study, casualties with PNI treated at US MTF were significantly more likely to survive than casualties treated at UK MTF, despite being equally likely to receive treatment. In addition, the odds of survival for casualties with PNI treated at Role 3 MTF were three times higher than those in a Role 2 MTF, despite being equally likely to receive treatment. This may reflect that Role 3 MTF were more likely to have surgeons formally trained in the treatment of PNI. We believe the results of this paper support previous multidisciplinary military consensus that neck exploration is an essential skill that must be retained by those surgeons deploying to coalition Role 2 and 3 MTF in future conflicts ^32^.

**Acknowledgements**

The authors would like to thank our colleagues at the UK Academic Department of Military Emergency Medicine and the US Joint Trauma Registry for their assistance in providing the data for this study.

**Contributor statement**

Planning: JB, DP

Conducting: JB, DP, JD, AB

Reporting: JB, DB, DP, LO, JBa, JC, JD

**Conflict of interest statement**

The authors have no declared conflict of interest in the collection and writing up of these results. Permission to publish has been granted by the US Department of Defense and the UK Ministry of Defence.

**Ethical approval**

Ethical approval was not required as this was a retrospective epidemiological study in which all data has been anonymized and no patient identifiable data included.

**Level of evidence**

Level 3: retrospective study with up to two negative criteria

**Funding statement**

The authors can confirm that no external sources of funding occurred with the production of this manuscript.

|  | US military treated in US MTF | UK military treated in UK MTF | Other coalition military treated in US MTF | Other coalition military treated in UK MTF | Host nation military treated in US MTF | Host nation military treated in UK MTF | Host nation civilians treated in US MTF | Host nation civilians treated in UK MTF |
| --- | --- | --- | --- | --- | --- | --- | --- | --- |
| Any body area | 28935 | 2013 | 3965 | 171 | 10681 | 527 | 19737 | 1557 |
| All neck | 1453 (5%) | 131 (7%) | 107 (3%) | 11 (6%) | 640 (6%) | 24 (5%) | 919 (5%) | 72 (5%) |
| Isolated neck | 696 (2%) | 26 (1%) | 74 (2%) | 1 (<1%) | 266 (2%) | 5 (1%) | 412 (2%) | 15 (1%) |
| Iraq | 1084 (75%) | 40 (31%) | 19 (18%) | 2 (18%) | 454 (71%) | 2 (8%) | 581 (63%) | 9 (13%) |
| Afghanistan | 369 (25%) | 91 (69%) | 85 (72%) | 9 (72%) | 186 (29%) | 22 (92%) | 338 (37%) | 63 (87%) |
| Male (%) | 1423 (98%) | 128 (98%) | 106 (100%) | 11 (100%) | 640 (100%) | 24 (100%) | 830 (90%) | 68 (4%) |
| Age, mean (range, S.D.) | 25.9 (18-57, 6.3) | 25.0 (18-53, 6.2) | 26.8 (18-48, 6.5) | 22.6 (20-31, 3.2) | 26.2 (17-55, 6.7) | 25.6 (19-36, 5.7) | 29.3 (1-74, 14.1) | 21.8 (4-55, 12.1) |
| ISS all neck injuries, mean (S.D) | 13.1 (11.9) | 19.0 (23.3) | 10.1 (11.5) | 8.9 (8.7) | 12.8 (10.9) | 16.8 (22.6) | 13.3 (10.9) | 14.0 (14.0) |
| ISS isolated neck injuries, mean (S.D.) | 6.9 (8.1) | 7.1 (15.3) | 6.8 (10.9) | 6.7 (8.1) | 7.3 (9.2) | 8.6 (14.9) | 8.2 (8.5) | 8.9 (7.1) |

**Table 1: Demographics of those casualties with neck injuries that survived to treatment at a Medical Treatment Facility (S.D.= Standard Deviation).**

| **Group** | **AIS 2005 diagnosis codes** | **US Military** | **UK military** | **Other Coalition Military** | Host nation **Military** | Host nation **Civilians** | **All** |
| --- | --- | --- | --- | --- | --- | --- | --- |
| **PENETRATING NECK INJURY** |  | 660 (100%) | 103 (100%) | 77 (100%) | 546 (100%) | 800 (100%) | 2186 (100%) |
| **Cervical skin/muscle damage** | 310099-310806, 316000-316006 | 542 (82%) | 99 (96%) | 53 (69%) | 373 (68%) | 548 (69%) | 1615 (74%) |
| **All cervical vessel injuries** | 320099, 320202-321099 | 223 (34%) | 20 (19%) | 14 (18%) | 119 (2%) | 179 (22%) | 555 (25%) |
| **Carotid artery injury** | 320202-320499 | 110 (17%) | 10 (10%) | 6 (8%) | 50 (9%) | 81 (10%) | 257 (12%) |
| **Internal jugular vein injury** | 320802-320899 | 61 (9%) | 9 (9%) | 2 (3%) | 45 (8%) | 57 (7%) | 174 (8%) |
| **Vertebral artery injury** | 321002-321099 | 32 (5%) | 5 (5%) | 3 (4%) | 19 (3%) | 24 (3%) | 83 (4%) |
| **Cervical tracheal injury** | 341602-341699 | 49 (7%) | 6 (6%) | 5 (6%) | 31 (6%) | 62 (8%) | 153 (7%) |
| **Cervical oesophageal injury** | 340102-340199 | 23 (3%) | 3 (3%) | 3 (4%) | 17 (3%) | 31 (4%) | 77 (4%) |
| **Laryngeal injury** | 340202-340299 | 69 (10%) | 9 (9%) | 11 (14%) | 40 (7%) | 56 (7%) | 185 (8%) |
| **Pharyngeal injury** | 340602-340699 | 79 (12%) | 7 (7%) | 8 (10%) | 27 (5%) | 57 (7%) | 178 (8%) |
| **Vagus/phrenic nerve injury** | 330099, 330299, 330499 | 13 (2%) | 2 (2%) | 1 (1%) | 5 (1%) | 6 (1%) | 27 (1%) |

**Table 2: Types of neck injuries found in those that survived to treatment at a Medical Treatment Facility. Percentages are those of Penetrating Neck Injury (PNI) only.**

| **Group** | **ICD 9 codes** | **OPCS-4 code** | **US Military** | **UK Military** | **Others US MTF** | **Others UK MTF** |
| --- | --- | --- | --- | --- | --- | --- |
| **NECK PROCEDURES (ALL)** |  |  | 626/1453 (43%) | 55/131 (42%) | 1563/1666 (94%) | 63/107 (59%) |
| **NECK PROCEDURES (PNI ONLY)** |  |  | 254/660 (38%) | 37/103 (36%) | 382/1325 (29%) | 36/86 (42%) |
| **Ligation or repair of neck vessel** | 06.92, 38.62, 38.81-39.32 | L29.8, L29.9, L30.1, L30.2 | 79/223 (35%) | 16/20 (80%) | 122/299 (41%) | 10/13 (75%) |
| **Ligation of neck vessel** | 06.92, 38.62, 38.81, 38.82 | L30.2 | 54/223 (24%) | 7/20 (35%) | 73/299 (24%) | 6/13 (75%) |
| **Repair of neck vessel** | 39.31, 39.32 | L29.8, L29.9, L30.1 | 25/223 (11%) | 9/20 (45%) | 49/299 (16%) | 4/13 (50%) |
| **Surgical tracheostomy** | 31.1, 31.12, 31.29, 31.74 | E42.1, E42.3 | 409 | 15 | 1247 | 37 |
| **Tracheal repair** | 31.71-31.73, 31.79 | E40.1-E40.3, | 22/49 (45%) | 1/6 (17%) | 61/91 (67%) | 7/7 (100%) |
| **Oesophageal repair** | 42.82, 42.89 | G07.4, G07.8 | 7/23 (30%) | 1/3 (34%) | 30/48 (63%) | 2/3 (67%) |
| **Laryngeal repair** | 30.1, 30.29, 30.3, 30.4, 31.61, 31.64, 31.69 | E31.8, E31.9 | 16/69 (23%) | 0/9 (0%) | 33/104 (32%) | 1/3 (33%) |
| **Pharyngeal repair** | 29.51, 29.53, 29.59, 29.99 | E21.4, E21.8, E21.9 | 17/79 (22%) | 0/7 (0%) | 27/91 (30%) | 0/1 (0%) |
| **Endovascular vessel treatment** | 39.72 | L31.3, L31.3, L31.4 | 6 | 0 | 4 | 0 |
| **Neck exploration/ debridement** |  |  |  | 52 |  | 53 |

**Table 3: Treatment performed for PNI in those that survived to treatment at a Medical Treatment Facility.**

| Relationship between death and neck wound according to DoDTR/JTTR | US military | UK military | Other treated in US MTF | Other treated in UK MTF |
| --- | --- | --- | --- | --- |
| All neck wounds (survivors + DoW) | 1453 | 131 | 1666 | 107 |
| DoW- neck wound present | 83 (6%) | 17 (13%) | 110 (7%) | 5 (5%) |
| DoW- neck wound contributory to death (AIS-6) | 7 (<1%) | 2 (2%) | 6 (<1%) | 1 (1%) |
| DoW- not related to neck wound | 25 (2%) | 15 (11%) | 21 (1%) | 4 (4%) |
| Unable to determine (survivors + DoW) | 51 (4%) | 0 (0%) | 83 (5%) | 0 (0%) |

**Table 4: Association between neck wound and fatalities based upon casualty cohort.**

**References**

1. Brennan J, Gibbons MD, Lopez M, Hayes D, Faulkner J, Eller RL, Barton C. Traumatic airway management in Operation Iraqi Freedom. *Otolaryngol Head Neck Surg*. 2011;144(3):376-380. doi:10.1177/0194599810392666

2. Breeze J, Allanson-Bailey LS, Hunt NC, Delaney RS, Hepper AE, Clasper J. Mortality and morbidity from combat neck injury. *J Trauma Acute Care Surg*. 2012;72(4):969-974. doi:10.1097/TA.0b013e31823e20a0

3. Clouse WD, Rasmussen TE, Peck MA, Eliason JL, Cox MW, Bowser AN, Jenkins DH, Smith DL, Rich NM. In-theater management of vascular injury: 2 years of the Balad Vascular Registry. *J Am Coll Surg*. 2007;204(4):625-632. doi:10.1016/j.jamcollsurg.2007.01.040

4. Kelly JF, Ritenour AE, McLaughlin DF, Bagg KA, Apodaca AN, Mallak CT, Pearse L, Lawnick MM, Champion HR, Wade CE, Holcomb JB. Injury severity and causes of death from Operation Iraqi Freedom and Operation Enduring Freedom: 2003-2004 versus 2006. *J Trauma*. 2008;64(2 Suppl):S21-6; discussion S26-7. doi:10.1097/TA.0b013e318160b9fb

5. Gellerfors M, Linde J, Gryth D. Helicopter in-flight resuscitation with freeze-dried plasma of a patient with a high-velocity gunshot wound to the neck in Afghanistan - A case report. *Prehosp Disaster Med*. 2015. doi:10.1017/S1049023X15005014

6. Breeze J, Gibbons AJ, Combes JG, Monaghan AM. Oral and maxillofacial surgical contribution to 21 months of operating theatre activity in Kandahar Field Hospital: 1 February 2007-31 October 2008. *Br J Oral Maxillofac Surg*. 2011;49(6):464-468. doi:10.1016/j.bjoms.2010.08.002

7. Breeze J, Gibbons AJ, Shieff C, Banfield G, Bryant DG, Midwinter MJ. Combat-related craniofacial and cervical injuries: a 5-year review from the British military. *J Trauma*. 2011;71(1):108-113. doi:10.1097/TA.0b013e318203304a

8. Breeze J, Monaghan AM, Williams MD, Clark RN, Gibbons AJ. Five months of surgery in the Multinational Field Hospital in Afghanistan with an emphasis on Oral and Maxillofacial injuries. *J R Army Med Corps*. 2013;156(2):125-128. doi:10.1136/jramc-156-02-15

9. Xydakis MS, Fravell MD, Nasser KE, Casler JD. Analysis of battlefield head and neck injuries in Iraq and Afghanistan. *Otolaryngol Head Neck Surg*. 2005;133(4):497-504. doi:10.1016/j.otohns.2005.07.003

10. Powers DB. Distribution of civilian and military maxillofacial surgical procedures performed in an Air Force theatre hospital: implications for training and readiness. *J R Army Med Corps*. 2010;156(2):117-121. doi:10.1136/jramc-156-02-13

11. Brennan J, Lopez M, Gibbons MD, Hayes D, Faulkner J, Dorlac WC, Barton C. Penetrating neck trauma in Operation Iraqi Freedom. *Otolaryngol Head Neck Surg*. 2011;144(2):180-185. doi:10.1177/0194599810391628

12. Brennan J. Experience of first deployed otolaryngology team in Operation Iraqi Freedom: the changing face of combat injuries. *Otolaryngol Head Neck Surg*. 2006;134(1):100-105. doi:10.1016/j.otohns.2005.10.008

13. Brennan J. Head and neck trauma in Iraq and Afghanistan: different war, different surgery, lessons learned. *Laryngoscope*. 2013;123(10):2411-2417. doi:10.1002/lary.24096

14. Schaefer SD. The treatment of acute external laryngeal injuries. “State of the art”. *Arch Otolaryngol Head Neck Surg*. 1991;117(1):35-39. doi:10.1001/archotol.1991.01870130041013

15. Rew DA, Clasper J, Kerr G. Surgical workload from an integrated UK field hospital during the 2003 Gulf conflict. *J R Army Med Corps*. 2004;150(2):99-106. doi:10.1136/jramc-150-02-06

16. Keller MW, Han PP, Galarneau MR, Brigger MT. Airway Management in Severe Combat Maxillofacial Trauma. *Otolaryngol Head Neck Surg*. 2015;153(4):532-537. doi:10.1177/0194599815576916

17. Wade AL, Dye JL, Mohrle CR, Galarneau MR. Head, face, and neck injuries during operation Iraqi Freedom II: Results from the Us Navy-Marine Corps Combat Trauma Registry. *J Trauma - Inj Infect Crit Care*. 2007;63(4):836-840. doi:10.1097/01.ta.0000251453.54663.66

18. Fox CJ, Gillespie DL, Weber MA, Cox MW, Hawksworth JS, Cryer CM, Rich NM, O'Donnell SD. Delayed evaluation of combat-related penetrating neck trauma. *J Vasc Surg*. 2006;44(1):86-93. doi:10.1016/j.jvs.2006.02.058

19. Rasmussen TE, Clouse WD, Jenkins DH, Peck MA, Eliason JL, Smith DL. Echelons of care and the management of wartime vascular injury: a report from the 332nd EMDG/Air Force Theater Hospital, Balad Air Base, Iraq. *Perspect Vasc Surg Endovasc Ther*. 2006;18(2):91-99. doi:10.1177/1531003506293374

20. Feldt BA, Salinas NL, Rasmussen TE, Brennan J. The joint facial and invasive neck trauma (J-FAINT) project, Iraq and Afghanistan 2003-2011. *Otolaryngol Head Neck Surg*. 2013;148(3):403-408. doi:10.1177/0194599817725713

21. Afghanistan troop numbers data: how many does each country send to the Nato mission there? *The Guardian*. https://www.theguardian.com/news/datablog/2009/sep/21/afghanistan-troop-numbers-nato-data. Published 2009.

22. Morrison JJ, Dubose JJ, Rasmussen TE, Midwinter MJ. Military Application of Tranexamic Acid in Trauma Emergency Resuscitation (MATTERs) Study. *Arch Surg*. 2012;147(2):113-119. doi:10.1001/archsurg.2011.287

23. Breeze J, Leason J, Gibb I, Allanson-Bailey L, Hunt N, Hepper A, Spencer P, Clasper J. Characterisation of explosive fragments injuring the neck. *Br J Oral Maxillofac Surg*. 2013;51(8):e263-6. doi:10.1016/j.bjoms.2013.08.005

24. Breeze J, Masterson L, Banfield G. Outcomes from penetrating ballistic cervical injury. *J R Army Med Corps*. 2012;158(2):96-100. doi:10.1136/jramc-158-02-05

25. Stannard A, Brown K, Benson C, Clasper J, Midwinter M, Tai NR. Outcome after vascular trauma in a deployed military trauma system. *Br J Surg*. 2011;98(2):228-234. doi:10.1002/bjs.7359

26. Patel JA, White JM, White PW, Rich NM, Rasmussen TE. A contemporary, 7-year analysis of vascular injury from the war in Afghanistan. *J Vasc Surg*. June 11, 2018.

27. Turner CA, Stockinger ZT, Gurney JM. Vascular surgery during U.S. combat operations from 2002 to 2016: Analysis of vascular procedures performed to inform military training. *J Trauma Acute Care Surg*. 2018;85(1S Suppl 2):S145-S153. doi:10.1097/TA.0000000000001849

28. Breeze J, Fryer R, Hare J, Delaney R, Hunt NC, Lewis EA, Clasper JC. Clinical and post mortem analysis of combat neck injury used to inform a novel coverage of armour tool. *Injury*. 2015;46(4):629-633. doi:10.1016/j.injury.2015.01.045

29. Breeze J. The problems of protecting the neck from combat wounds. *J R Army Med Corps*. 2010;156(3):137-138. doi:10.1136/jramc-156-03-01

30. Tyler JA, Clive KS, White CE, Beekley AC, Blackbourne LH. Current US military operations and implications for military surgical training. *J Am Coll Surg*. 2010;211(5):658-662. doi:10.1016/j.jamcollsurg.2010.07.009

31. Turner CA, Stockinger ZT, Gurney JM. Combat surgical workload in Operation Iraqi Freedom and Operation Enduring Freedom: The definitive analysis. *J Trauma Acute Care Surg*. 2017;83(1):77-83. doi:10.1097/TA.0000000000001496

32. Breeze J, Blanch R, Baden J, Monaghan AM, Evriviades D, Harrisson SE, Roberts S, Gibson A, MacKenzie N, Baxter D, et al. Skill sets required for the management of military head, face and neck trauma: a multidisciplinary consensus statement. *J R Army Med Corps*. 2018;164(2):133-138. doi:10.1136/jramc-2017-000881

**List of tables in print**

Table 1: Demographics of those casualties with neck injuries that survived to treatment at a Medical Treatment Facility (S.D.= Standard Deviation).

Table 2: Types of neck injuries found in those casualties that survived to treatment at a Medical Treatment Facility. Percentages are those of Penetrating Neck Injury (PNI) only.

Table 3: Treatment performed for PNI in those that survived to treatment at a Medical Treatment Facility.

Table 4: Association between neck wound and fatalities based upon casualty cohort.

**List of figures**

Figure 1: CONSORT flow diagram demonstrating inclusion and exclusion criteria for those who survived to treatment at a Medical Treatment Facility.

Figure 2: Comparison of treatment performed at different Medical Treatment Facilities for casualties with neck injuries that survived to treatment at a Medical Treatment Facility.
